# Inter-observer variability of ^90^Y PET/CT dosimetry in hepatocellular carcinoma after glass microspheres transarterial radioembolization

**DOI:** 10.1186/s40658-020-00302-1

**Published:** 2020-05-12

**Authors:** Nicolas Meyers, Alexandre Jadoul, Claire Bernard, Jean Delwaide, Anne Lamproye, Olivier Detry, Pierre Honoré, Laurent Gerard, Roland Hustinx

**Affiliations:** 1 Division of Nuclear Medicine and Oncological Imaging, CHU de Liege, University Hospital of Liege, B35 Domaine Universitaire du Sart-Tilman, 4000 Liege, Belgium; 2 Division of Hepato-Gastroenterology and Digestive Oncology, University Hospital of Liege, Liege, Belgium; 3 Division of Abdominal Surgery and Transplantation, University Hospital of Liege, Liege, Belgium; 4 Division of Radiology, University Hospital of Liege, Liege, Belgium

**Keywords:** Radioembolization, Hepatocarcinoma, Dosimetry, Microspheres, Reproducibility

## Abstract

**Introduction:**

Strong correlation has been demonstrated between tumor dose and response and between healthy liver dose and side effects. Individualized dosimetry is increasingly recommended in the current clinical routine. However, hepatic and tumor segmentations could be complex in some cases. The aim of this study is to assess the reproducibility of the tumoral and non-tumoral liver dosimetry in selective internal radiation therapy (SIRT).

**Material and methods:**

Twenty-three patients with hepatocellular carcinoma (HCC) who underwent SIRT with glass microspheres were retrospectively included in the study. Tumor (TV) and total liver volumes (TLV), and mean absorbed doses in tumoral liver (TD) and non-tumoral liver (THLD) were determined on the ^90^Y PET/CT studies using Simplicit90YTM software, by three independent observers. Dosimetry datasets were obtained by a medical physicist helped by a nuclear medicine (NM) physician with 10 years of experience (A), by a NM physician with 4-year experience (B), and by a resident who first performed 10 dosimetry assessments as a training (C). Inter-observer agreement was evaluated using intra-class correlation coefficients (ICC), coefficients of variation (CV), Bland-Altman plots, and reproducibility coefficient (RDC).

**Results:**

A strong agreement was observed between all three readers for estimating TLV (ICC 0.98) and THLD (ICC 0.97). Agreement was lower for TV delineation (ICC 0.94) and particularly for TD (ICC 0.73), especially for the highest values. Regarding TD, the CV (%) was 26.5, 26.9, and 20.2 between observers A and B, A and C, and B and C, respectively, and the RDC was 1.5. Regarding THLD, it was 8.5, 12.7, and 9.4, and the RDC was 1.3.

**Conclusion:**

Using a standardized methodology, and regardless of the different experiences of the observers, the estimation of THLD is highly reproducible. Although the reproducibility of the assessment of tumor irradiation is overall quite high, large variations may be observed in a limited number of patients.

## Introduction

Selective internal radiation therapy (SIRT) with glass or resin microspheres has been used [1–4] in the treatment of liver tumors for over two decades. However, SIRT is currently not recommended as a first-line treatment in guidelines [5], as recent randomized multi-centric studies in HCC and colorectal metastases failed to establish its clinical role [6–8]. A major limitation of these trials was the use of empirical dosimetry, which assumes a homogeneous distribution of the injected activity in the liver [9, 10]. Over the past decade, correlations have been established between tumor dose and response, on the one hand, and between healthy liver dose and side effects, on the other hand [11, 12]. Individualized dosimetry is thus increasingly advocated in current clinical routine.

A key step in the dosimetry process is the identification and the segmentation of the target volumes. This may prove highly complex, especially in case of multifocal HCC or diffuse metastatic disease, and may generate an inter-observer variability of volumes measurements and consequently of estimated doses. Quite surprisingly, very limited data is available on this specific topic. Monsky et al. [13] showed a strong inter-observer agreement both for delineating tumoral and non-tumoral volumes. However, the study focused on a new semi-automated segmentation algorithm and there was no report on the dosimetry. The only research investigating dose measurements reported a clinically acceptable inter-observer variability for healthy liver but larger fluctuations for assessing the dose to neoplastic lesions [14].

The aim of the present study is therefore to assess the reproducibility of the tumoral and non-tumoral liver dosimetry in SIRT, on the basis of post-treatment Yttrium-90 (^90^Y) combined positron emission tomography-computed tomography (PET/CT) images.

## Material and methods

### Patients

Twenty-three patients with HCC who underwent SIRT with glass microspheres (TheraSpheres®) between 2016 and 2019 were retrospectively included in our study. Two of them underwent a second radioembolization, leading to a total of 25 treatments with an average injected activity of 1.98 ± 1.23 GBq. All patients were treated according to the treatment algorithms applied in our institution and after discussion during our multidisciplinary tumor board. In the six cases with multiple lesions, the overall mean tumor dose was considered. The mean age of the cohort was 66 ± 9 years (min 48 and max 84) for a male/female ratio of 1.7.

Patient characteristics are summarized in Table 1.

**Table 1:** Summary of patients characteristics

Categories	*N*	Mean ± SD	Number (percent)	Median (min-max)
Age (years)	25	66.2 ± 9.05		
Sex	25			
F			9 (36)	
H			16 (64)	
Average injected activity (GBq)		1.98 ± 1.23		
Tumor size (ml)				77.4 (10.6–1017.5)
Interval between CE-CT and ^90^Y PET/CT (days)		24 ± 7		28 (1–30)

### ^90^Y PET/CT

PET/CT studies were performed in our institution, no later than 24 h after the treatment, using a Big Bore or a Gemini TF 16 PET/CT system (both Philips Medical Systems, Cleveland, OH, USA). Three steps of 13 min per bed position (axial field of view (FOV) of 18 cm) were acquired, with an overlap of 50%. All acquisitions were corrected for scatter, decay, attenuation, and random events. Attenuation correction was performed using a low-dose computed tomography (CT) (120 kV and 50 mAs or 80 mAs, depending on the patient weight). For reconstruction, we used an iterative algorithm OSEM list-mode incorporating time of flight with 4 iterations and 8 subsets. The voxel size was 4 × 4 × 4 mm for PET images and 1.17 × 1.17 × 3 mm for low-dose CTs. The CT matrix was 512 and those of the PET 169 or 144 depending on the field of view acquisition dimension.

### Hepatic segmentation and dosimetry

Tumor and non-tumor volumes were measured using both the ^90^Y PET/CT and a three-phase injected CT that was performed for primary staging within 1 month prior to treatment. The mean absorbed doses in the tumoral liver (TD) and non-tumoral liver (THLD) were determined using Simplicit90YTM (version 2.1) software (MIRADA Medical ®) by three sets of independent observers.

One dataset was obtained as part of the daily clinical practice by a medical physicist helped by a nuclear medicine (NM) physician with 10 years of experience in SIRT (A). The second was performed by a NM physician with 4-year experience (B). The third was performed by a resident with limited experience and who first performed 10 dosimetry assessments as a training (C). This training set is not included in the current study. The time needed for the process was not precisely recorded but there were no major variations among the 3 operators in this regard.

The tumor volumes were manually delineated slice-by-slice on the three-phase injected CT, transposed to ^90^Y PET/CT, and adapted to correct for rigid misregistration and volume variations between contrast-enhanced (CE) CT and SIRT. Such manual adaptation was required in all cases. Non-enhancing parts of lesions were included in the tumoral volume. In the subjects with multiple lesions, each tumor volume was similarly delineated on the CE-CT, i.e., manually and slice-by-slice. The sum of these volumes led to the total tumoral volume, which was used to calculate the mean TD, without including any normal tissue. Total liver volume was delineated using the automated segmentation algorithm provided in the software, on the CT part of the PET/CT, and imperfections were manually rectified. The volume of injected liver, corresponding to the portion of the liver that has received microspheres, was determined on the PET images using a variable thresholding, to match as much as possible the CT limits. Non-tumoral liver volume was obtained by subtracting the tumoral volume from the total liver volume. Lung shunt fraction was arbitrarily set at 0%, this value having no influence on variability.

The following parameters were obtained for each patient:

- Tumoral volume (TV)
- Tumoral dose (TD)
- Total liver volume (TLV)
- Total healthy liver dose (THLD)

### Statistics

The continuous data were tested for normality (Shapiro-Wilk test). For the normally distributed value (TLV), paired Student’s *t* test and ANOVA-2 test were used to compare the average values between the readers. Non-parametric tests (Wilcoxon signed-rank and Friedman tests) were performed to compare the other parameters. Agreement was measured by the intra-class correlation coefficient (ICC), by Bland’s and Altman’s graphs, and by the coefficient of variation (CV). Variability was also assessed by the reproducibility coefficient (RDC) as defined by Haste et al. [14]. The RDC represents the maximum ratio of doses values obtained from the three different reviewers in 95% of cases, with a 95% confidence interval (95%CI). The results are considered significant at the uncertainty level of 5% (*p* < 0.05). Calculations were made using SAS version 9.4 and graphics using R version 3.5, except the RDC, for which a user-written routine was developed (Additional file 1).

## Results

### Non-tumoral liver

A strong agreement was observed between all three readers for estimating the total liver volume with an ICC (95%CI) of 0.98 (0.97–0.99). Neither the paired *t* tests nor the ANOVA-2 showed any statistically significant differences between mean volumes (1749 ± 507 ml, 1748 ± 482 ml, and 1735 ± 496 ml for observers A, B, and C, respectively). This was confirmed by the low values of CV (5.1% between A and B, 3.9% between A and C, and 2.6% between B and C)

**Fig. 1:**
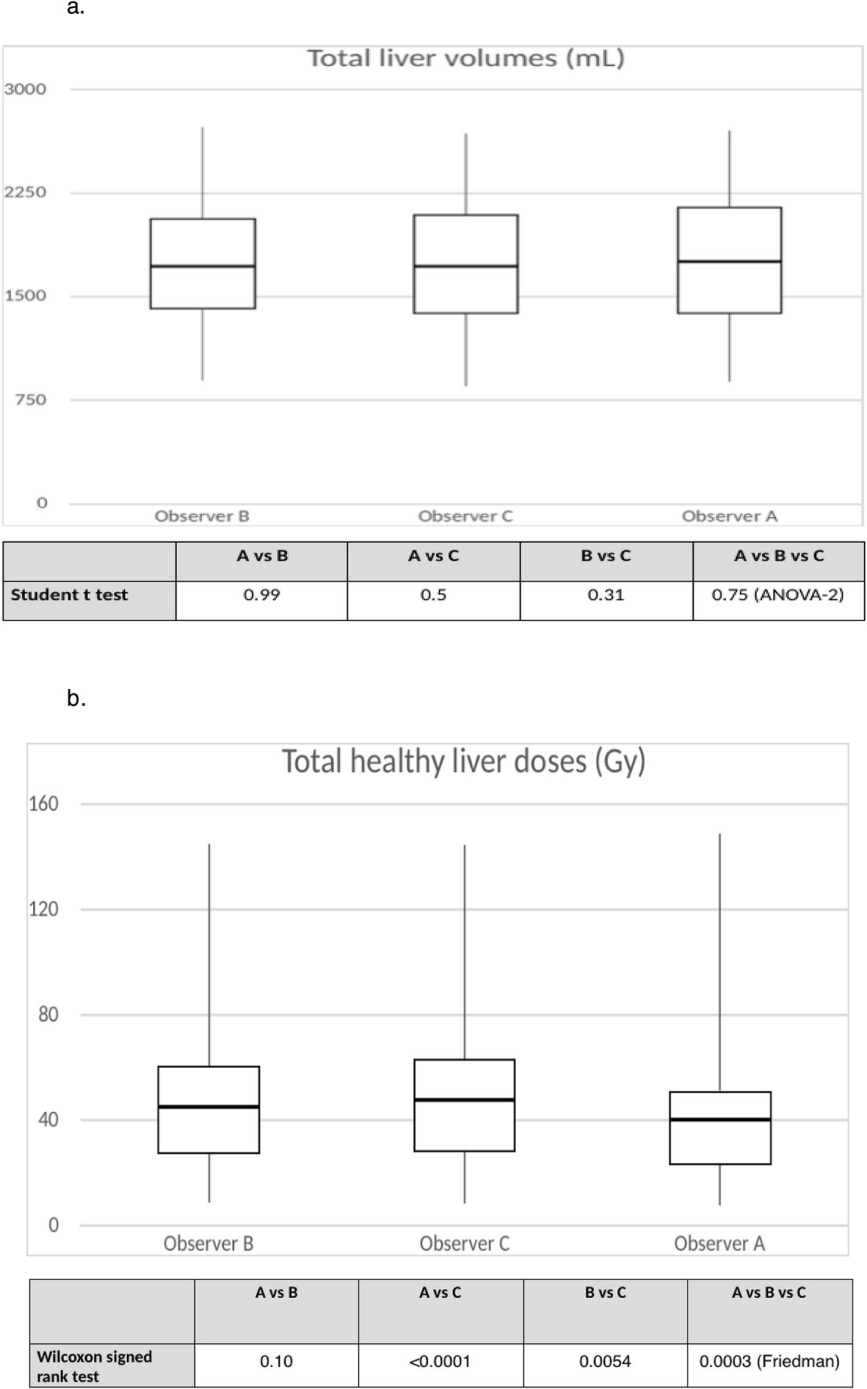
**a***B*ox plots for non-tumoral volumes and *p* values from *t* test and ANOVA. The volume in milliliters is plotted on the *y*-axis, and the observer is plotted on the *x*-axis. Upper horizontal line of box represents the 75th percentile; lower horizontal line of the box, 25th percentile; horizontal bar within box, median; upper extremity of line outside the box, maximum value; and lower extremity of line outside the box, minimum value. **b** Box plots for non-tumoral doses and *p* values from Wilcoxon signed-rank and Friedman tests. The dose in grays is plotted on the *y*-axis, and the observer is plotted on the *x*-axis. Upper horizontal line of box represents the 75th percentile; lower horizontal line of the box, 25th percentile; horizontal bar within box, median; upper extremity of line outside the box, maximum value; and lower extremity of line outside the box, minimum value

**Fig. 2:**
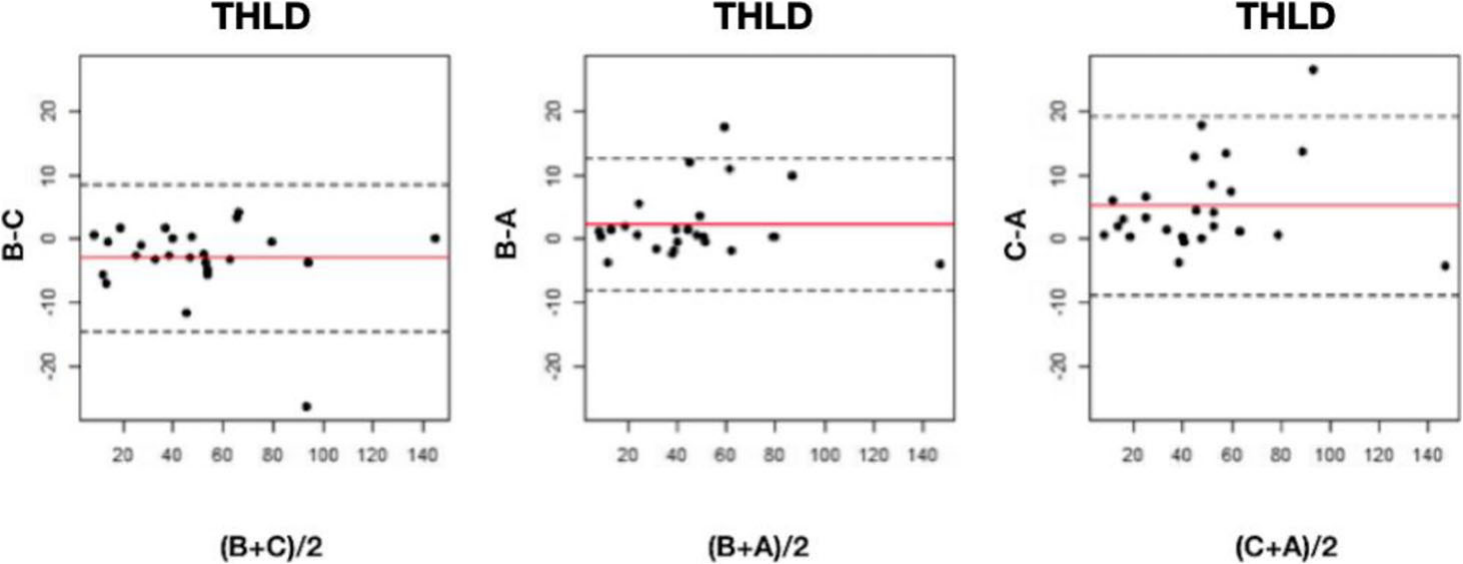
Bland-Altman graph for non-tumoral doses (THLD) in grays. The red line represents the average of the differences between observers and the dotted line, the mean of the differences ± 2 standard deviations

The mean healthy liver doses were 45.3 ± 30.2 Gy for observer A, 47.5 ± 30.5 Gy for observer B, and 50.5 ± 31.5 Gy for observer C. Differences between readers were statistically significant overall (*p* = 0.0003) and between readers A and C (*p* < 0.0001) and B and C (*p* = 0.0054). The observations for non-tumoral volumes and doses are represented in box plot diagrams (Fig. 1). The ICC for doses between the 3 readers was 0.97 (95%CI 0.96–0.99), and 0.98 (95%CI 0.97–0.99) between readers A and B, 0.96 (95%CI 0.88–0.99) between A and C, and 0.98 (95%CI 0.96–0.99) between B and C. As shown in the Bland-Altman plots (Fig. 2), a difference greater than 20 Gy occurred twice. The RDC for THLD was 1.33 (95%CI 1.26–1.68).

### Tumor

**Fig. 3:**
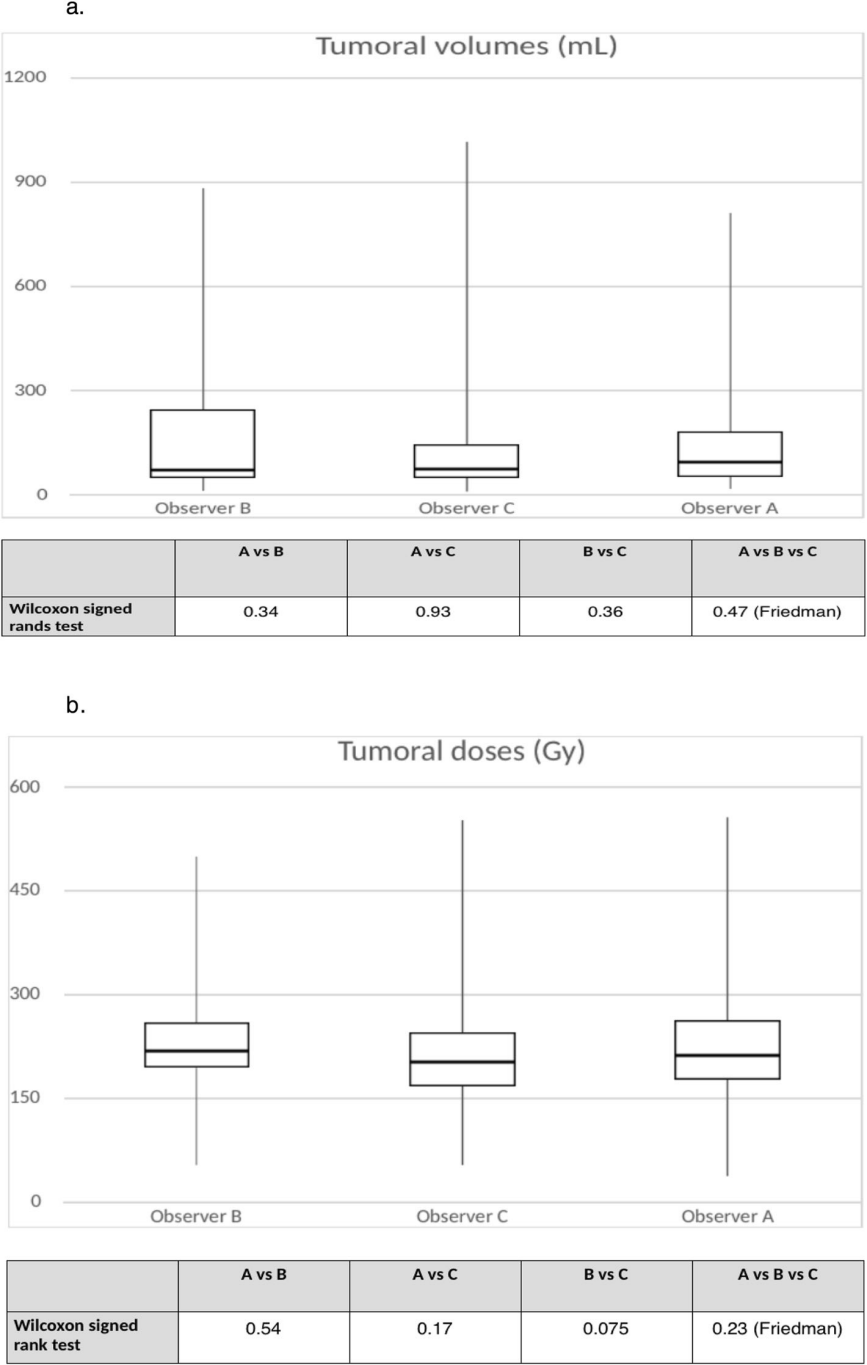
**a** Box plots for tumoral volumes and *p* values from Wilcoxon signed-rank and Friedman tests. The volume in milliliters is plotted on the *y*-axis, and the observer is plotted on the *x*-axis. Upper horizontal line of box represents the 75th percentile; lower horizontal line of the box, 25th percentile; horizontal bar within box, median; upper extremity of line outside the box, maximum value; and lower extremity of line outside the box, minimum value. **b** Box plots for tumoral doses and *p* values from Wilcoxon signed-rank and Friedman tests. The dose in grays is plotted on the *y*-axis, and the observer is plotted on the *x*-axis. Upper horizontal line of box represents the 75th percentile; lower horizontal line of the box, 25th percentile; horizontal bar within box, median; upper extremity of line outside the box, maximum value; and lower extremity of line outside the box, minimum value

**Fig. 4:**
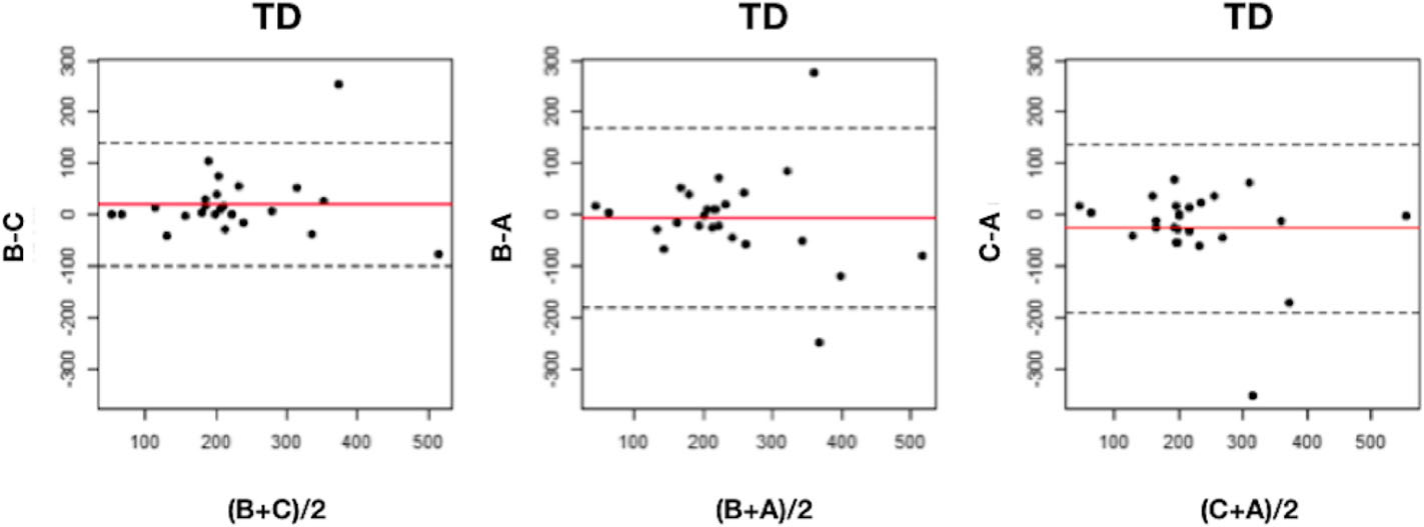
Bland-Altman graph for tumoral doses (TD) in grays. The red line represents the average of the differences between observers and the dotted line, the mean of the differences ± 2 standard deviations

Regarding the tumor volume delineation, the ICC was 0.94 (95%CI 0.92–0.97) between all three readers. No significant difference was observed by Wilcoxon signed-rank and Friedman tests when comparing the mean volumes (179 ± 208 ml (median 94, min 17, max 813), 199 ± 258 ml (median 77, min 12, max 890), and 185 ± 265 ml (median 76, min 11, max 1017) for observers A, B, and C, respectively). CV was 35.9%, 36.8%, and 25.5% between A and B, A and C, and B and C, respectively. Regarding the TD obtained by the various readers, Wilcoxon signed-rank and Friedman tests did not show statistically significant differences either. The observations for tumoral volumes and doses are represented in box plot diagrams (Fig. 3). Nevertheless, ICC was lower than for THLD: 0.70 (95%CI 0.49–0.85), 0.71 (95%CI 0.51–0.86), and 0.81 (95%CI 0.68–0.91) between A and B, A and C, and B and C, respectively, and 0.73 (95%CI 0.64–0.86) between all three readers. Bland-Altman analysis is shown in Fig. 4. Dispersion was larger in lesions that received a dose greater than 300 Gy. The RDC for TD was 1.52 (95%CI 1.38–2.25).

**Fig. 5:**
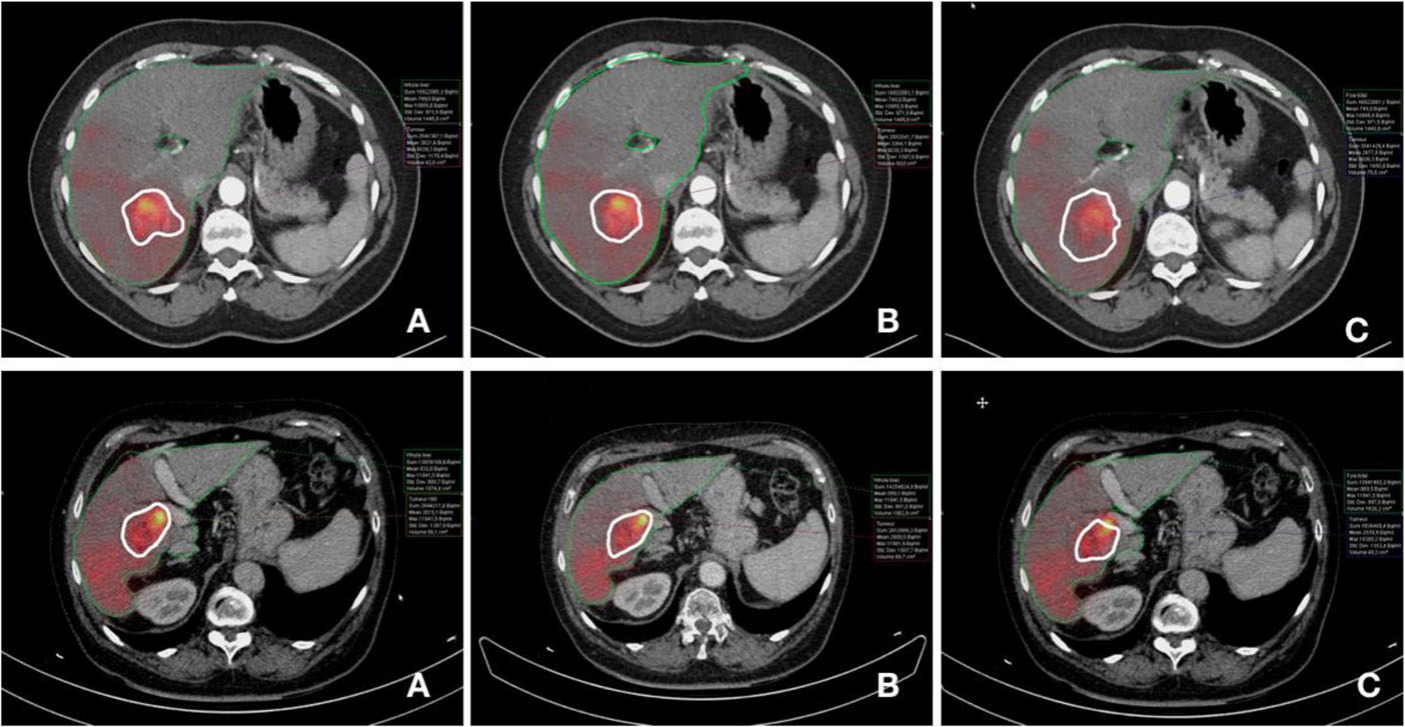
Example of tumor volume delineation in two patients. **A**, **B**, and **C** are the observers. The upper row shows a tumor with differences in the contouring of the volume, whereas the differences are more limited in the patient represented in the lower row

Figure 5 shows an example of volume delineation.

The results are summarized in Tables 2 and 3.

**Table 2:** Summary of agreement test results for TLV, THLD, TV, and TD

	A vs B	A vs C	B vs C	A vs B vs C
**Total liver volumes**				
**ICC (95%CI)**	0.967 (0.945–0.985)	0.981 (0.977–0.984)	0.992 (0.986–0.996)	0.980 (0.973–0.990)
**CV (%)**	5.13	3.93	2.56	
**Total healthy liver doses**				
**ICC (95%CI)**	0.983 (0.969–0.993)	0.961 (0.877–0.986)	0.978 (0.955–0.991)	0.974 (0.956–0.989)
**CV (%)**	8.5	12.7	9.4	
**Tumor volumes**				
**ICC (95%CI)**	0.917 (0.861–0.962)	0.921 (0.868–0.964)	0.965 (0.941–0.984)	0.936 (0.915–0.969)
**CV (%)**	35.9	36.8	25.5	
**Tumor doses**				
**ICC (95%CI)**	0.695 (0.494–0.853)	0.706 (0.512–0.858)	0.814 (0.684–0.913)	0.734 (0.644–0.861)
**CV (%)**	26.5	26.9	20.2

**Table 3:** Summary of mean values ± SD of TLV, THLD, TV, and TD

	Mean ± SD A	Mean ± SD B	Mean ± SD C
**Total liver volumes (ml)**	1749 ± 507	1748 ± 482	1735 ± 496
**Total healthy liver doses (Gy)**	45.3 ± 30.2	47.5 ± 30.5	50.5 ± 31.5
**Tumor volumes (ml)**	179 ± 208	199 ± 258	185 ± 265
**Tumor doses (Gy)**	238 ± 120	233 ± 106	212 ± 100

### Target dose

Considering a target dose > 200 Gy [15–17] for the tumor and < 50 Gy [18] for the healthy liver, 7/25 (28%) patients (tumor) and 2/25 (8%) patients (non-tumor) would have been classified by the various observers in different categories, i.e., target reached/not reached. Considering a target dose < 75 Gy for non-tumoral liver, observers agreed on the category for all patients (0% classified in different categories).

## Discussion

Relationship between response and tumor dose is now well established in liver tumors [15]. However, considering ^90^Y PET/CT-based dosimetry in HCC, data are limited and there remain discrepancies between studies in determining the efficient tumoral threshold dose. Song et al. [16] reported a longer PFS with a TD > 200 Gy, while Kao et al. observed a complete radiological response with a minimum dose to 70% tumor volume > 100 Gy [19]. Furthermore, a significantly higher median TD was observed in responders (225 Gy compared to 83 Gy in non-responders) in a recent prospective trial in a cohort of 27 patients [17]. In the latter study, a dose threshold of 200 Gy was 100% specific and had a 100% positive predictive value whereas all non-responders received a dose < 200 Gy. On the other hand, Srinivas et al. did not show a statistically significant relationship between TD and radiological response [20]. Considering healthy liver, this study showed an association between THLD and the presence of two or more radiation-induced complications. Another prospective work conducted by Chan et al. demonstrated that the likelihood of toxicity exceeds 50% at a THLD threshold of 54 Gy [18].

In any case, a personalized dosimetry seems currently essential to optimize clinical results of SIRT [21] as factors such as small sample size, differences in activity calculation methods, response assessment criteria, microsphere type, and study design all contribute to some extent to explain the heterogeneity of the published data. Further, the way to delineate tumoral and non-tumoral liver may be subject to inter-observer variability and can potentially generate changes in TD and THLD between different physicians.

Although our results suggest that the delineation of non-tumoral volumes and the estimation of corresponding doses are reproducible, statistical comparison of samples showed significant differences of THLD between observers A-C and B-C, explained by the fact that this parameter is also influenced by the manual delineation of the tumor. The slightly lower ICC between A-C and B-C compared to A-B may be related to the lower experience of observer C. Such effect appears limited, however, given the overall high ICC, low CV, and more essentially the Bland-Altman analysis that all point toward a high reproducibility. Furthermore, it appears that all patients except 2 (8%) were classified as having received a THLD lower than 50 Gy by all three observers. This would mean that the threshold that used to be well accepted to avoid toxicity can be individually assessed with a great reproducibility [22]. If we consider a THLD threshold at 75 Gy, which was recently advocated by international experts [21], all observers classified every patient in the same category.

Conversely, despite the high ICC values for TV and although the analysis of mean TD values did not show statistically significant differences between observers, all other variables point toward a greater variability in particular an ICC of 0.70 and a greater dispersion in the Bland-Altman plots, probably due to the fact that correction for misregistration between contrast-enhanced CT and ^90^Y PET/CT may differ between observers. From a clinical perspective and considering 200 Gy as the target, 28% of the patients were classified in different groups depending on who performed the dosimetry. In addition to the limitations recently reported regarding the predictive value of the dosimetry performed on the ^99m^Tc-macroaggregated albumin (MAA) single-photon emission computed tomography (SPECT/CT) with regard to the actual dose delivered to the tumor, this emphasizes the need for further work for harmonizing the tumor dosimetry, in order to eventually optimize tumor response [15, 23–25]. On the other hand, it appears that current methodologies appear quite reliable for predicting and assessing the healthy liver dose, which is highly relevant for preventing toxicity.

Regarding hepatic volume segmentation, we demonstrated high agreement both for TV and TLV, comparable to that obtained by Monsky et al. [14]. Although they used a semi-automated segmentation software, their ICC values (with measurements repeated twice) were similar to ours: 0.98 and 0.99 for TLV, in comparison with 0.98 between all three readers in our study, and 0.98 and 0.99 for TV when we reached 0.94.

Our dosimetry results are overall in line with the only study aimed at addressing this question [13]. Haste et al. indeed observed a better reproducibility for TLHD compared to TD estimation, in a cohort of 73 HCC patients. To assess the variability, they used reproducibility coefficient (RDC), which represents the maximum ratio of doses values obtained from observers in 95% of cases, and obtained values of 1.4 (95%CI 1.3–1.6) for THLD and 1.6 (95%CI 1.5, 1.8) for TD. Our results appear comparable, both for THLD, with a RDC of 1.3 (95%CI 1.3–1.7) and for TD, with a value of 1.5 (95%CI 1.4–2.2). Furthermore, our results were obtained in 3 sets of readers with various levels of experience.

In addition, taking into account the strong correlation between ^99m^Tc-MAA SPECT/CT and ^90^Y PET/CT in HCC in non-tumoral liver [26], we may infer a clinically meaningful translation of these results in greatly reducing the risk of radiation-induced liver disease in a reliable and reproducible fashion. One should be more careful in predicting the effectiveness of SIRT based upon the tumor dosimetry.

Of note, the experience of the various operators did not significantly alter the reproducibility of the dosimetry, but a learning curve had been allocated to the least experienced one, and the procedure was performed in a controlled environment, using a well-defined methodology. In the future, semi-automated determination of tumoral and healthy liver could further help to standardize the methodology for hepatic segmentation [14, 27]. Hence, a consensus on TD and THLD goals would be easier to find between different centers.

## Conclusion

Accurate estimation of post-treatment TD and THLD is a central element to predict therapeutic efficacy of SIRT. Using a standardized methodology, ^90^Y PET/CT dosimetry in HCC after glass microspheres is strongly reproducible in healthy liver, even with different levels of experience between observers, which is highly relevant in the clinical practice for preventing potential toxicity. On the other hand, despite a fairly good agreement between observers, caution should be exercised for tumoral lesions as individual variations may be important, especially for the highest TD.

## Supplementary information

**Additional file 1.** User-written SAS script used to compute the RDC.

## Data Availability

The datasets used and/or analyzed during the current study are available from the corresponding author on reasonable request.

## Abbreviations

CE: Contrast-enhanced
CV: Coefficients of variation
CT: Computed tomography
FOV: Field of view
ICC: Intra-class correlation coefficients
MAA: Macroaggregated albumin
NM: Nuclear medicine
PET: Combined positron emission tomography-computed tomography
RDC: Reproducibility coefficient
SIRT: Selective internal radiation therapy
SPECT/CT: Single-photon emission computed tomography
TD: Mean absorbed doses in tumoral liver
THLD: Mean absorbed doses in non-tumoral liver
TLV: Total liver volumes
TV: Tumor volumes
^90^Y: Yttrium-90
