# Supplementary material for: Inter-observer variability of 90Y PET/CT dosimetry in hepatocellular carcinoma after glass microspheres transarterial radioembolization
Source: EJNMMI Phys. 2020 May 12;7:29. doi: 10.1186/s40658-020-00302-1 (PMC7218042; doi:10.1186/s40658-020-00302-1)
Supplement: Supplementary file 1 — Additional file 1. User-written SAS script used to compute the RDC. [file 40658_2020_302_MOESM1_ESM.pdf]

Appendix 1 : User-written SAS script used to compute the RDC.

As there is no SAS command to calculate the RDC, an “in house” SAS script was written based on the article of Haste et al [14] :

- For any given patient and any given parameter (e.g. liver volume), the data consist of three measurements made by the three physicians (X1, X2, X3).
- Then, we calculate the 6 possible ratios of these 3 measurements, specifically: X1/X2, X2/X1, X1/X3, X3/X1, X2/X3 and X3/X2.
- Thus, for the n patients, we have a total of N = 6 x n ratios.
- The RDC (according to Haste et al.) corresponds to the 95th percentile (P95) of the distribution of the N ratios. Thus it is necessary to rank all the N ratios.
- The 95% confidence interval (95%CI) for RDC is then determined as follows:

o The lower limit of the 95%CI is the ratio with rank “r”, where r is the greatest integer $\leq N \times 0.95 + 0.5 - 1.96 \times \sqrt{N \times 0.95 \times 0.05}$

o The upper limit of the 95%CI is the ratio with rank “s”, where s is the smallest integer $\geq N \times 0.95 + 0.5 + 1.96 \times \sqrt{N \times 0.95 \times 0.05}$
